# “Poor Effort” Does Not Account for Reduced Forced Vital Capacity in Asthmatic Children

**DOI:** 10.3389/fped.2021.596384

**Published:** 2021-05-25

**Authors:** Yong Feng, Que Yang, Yunxiao Shang

**Affiliations:** Department of Pediatrics, Shengjing Hospital of China Medical University, Shenyang, China

**Keywords:** asthma, children, forced vital capacity, exhalation time, quality control, small airway dysfunction

## Abstract

**Purpose:** Poor forced vital capacity (FVC) effort has been considered to be the main reason for FVC reduction by the ATS/ERS guideline; however, this has rarely been mentioned in previous studies. The present study aims to determine whether reduced FVC in asthmatic children is correlated to poor FVC effort.

**Methods:** A total of 209 asthmatic children within 5–13 years old were included and divided into reduced FVC (“restricted,” *n* = 66) and typical obstruction group (“obstructed,” *n* = 143). Forced expiratory flows before and after bronchodilation were recorded in asthmatic children. The differences in clinical characteristics, spirometric results, FVC effort, and bronchodilator response were compared between two groups. Exhalation time (ET) was divided into effective ET (ETe) and plateau ET (ETp) by the start point of exhalation plateau on the time-volume curve. FVC effort was assessed by ET, ETp, and back extrapolated volume (EV)/FVC (%).

**Results:** Asthmatic children in the restricted group had significantly higher slow vital capacity (SVC)/FVC (%), higher EV/FVC (%), shorter ET, shorter ETe, and longer ETp, when compared with those with obstructed. In the obstructed group, ET (*r* = 0.201, *P* = 0.016) and ETe (*r* = 0.496, *P* < 0.001) positively correlated with FVC, and ETp (*r* = −0.224, *P* = 0.007) negatively correlated with FVC. In the restricted group, FVC positively correlated with ETe (*r* = 0.350, *P* = 0.004) but not ET and ETp. FVC z-score significantly correlated with total IgE (*n* = 51, *r* = −0.349, *P* = 0.012) and with FEF_25−75%_ z-score (*n* = 66, *r* = 0.531, *P* < 0.001) in the restricted group. The further logistic regression revealed that the risk of restricted increased by 1.12 (95% CI, 1.04–1.22, *P* = 0.005) with every 1% increase in %ΔFVC. In subjects with restricted and bronchodilation tests, %ΔFVC was significantly associated with FeNO (*n* = 29, *r* = 0.386, *P* = 0.039), FEF_25−75%_ z-score (*n* = 29, *r* = −0.472, *P* = 0.010), and SVC/FVC (%) (*n* = 19, *r* = 0.477, *P* = 0.039) but not with EV/FVC (%), ET, ETe, or ETp (*P* > 0.05).

**Conclusion:** These findings suggested that “poor FVC effort” does not account for the FVC reduction in asthmatic children. Short ET and high SVC/FVC (%) are characteristics of reduced FVC.

## Introduction

Asthma is characterized by variable expiratory airflow limitation, which is caused by airway smooth muscle contraction, airway edema, airway thickening, or mucus hypersecretion. This airflow limitation is best documented by lung function tests, with a reduced ratio of forced expiratory volume in 1 s (FEV_1_) to forced vital capacity (FVC) (FEV_1_/FVC) from spirometry (1), especially the obstruction of the small airways, which can be revealed by the reduced mean forced expiratory flow between 25 and 75% of FVC (FEF_25−75%_) in spirometry (2, 3). Once the obstructive defect (reduced FEV_1_/FVC) is confirmed, the variation in airflow limitation is assessed from the variation in FEV_1_, while FVC and total lung capacity (TLC) are rarely mentioned in present asthma guidelines (4–6). FVC decreases in some phenotypes of asthma. However, it has been suggested that FVC reduction might not be due to a restrictive pattern when TLC and FEV_1_/FVC are within the normal range and might be caused by an elevated residual volume (RV)/TLC ratio, which is thought to be a special obstructive defect (7–10). This special obstructive defect has also been termed as pseudo-restrictive defect (7), small airway obstructive syndrome (11), or non-specific pattern (NSP) (8) in different studies.

The underlying pathophysiology mechanisms for lung volume changes in asthma are not well-described. Air trapping and premature distal airway closure assessed by increased RV/TLC caused by severe asthma and unstable asthma have been considered the main reasons (7, 12). However, the American Thoracic Society and European Respiratory Society (ATS/ERS) guidelines for lung function test interpretation suggests that this pattern frequently reflects the failure of a patient to completely inhale or exhale, leading to increased RV and normal TLC (1). Furthermore, few studies have evaluated the exhalation times (ET) to identify the FVC effort, although the maneuvers in these studies met the test criteria (7–10). It is known that some children have a short attention span and are easily distracted, leading to short ET. The short ET may meet the standard set by the ATS/ERS but is not the best, which still suggest a poor FVC effort. The contribution of poor FVC effort to reduced FVC remains unclear in asthmatic children. Consequently, the aim of the present study was to determine whether reduced FVC is associated with poor FVC effort.

## Methods

### Subjects

A retrospective analysis was conducted on subjects studied in our Pulmonary Function Laboratory between 2015 and 2018. The selection criteria were as follows: subjects <14 years old, subjects who visited the hospital for routine follow-up of asthma disease, and subjects who had eligible spirometry results with the pattern of “restricted” or “obstructed” defined below. The diagnosis of asthma was made by physician based on the Global Initiative for Asthma guidelines (13) and obtained from medical records. Children were excluded when they had other diagnoses of chronic disease than asthma, such as cystic fibrosis, bronchopulmonary dysplasia, scoliosis, or restrictive lung disease. Electronic medical records were examined for the course of disease, treatment duration, exacerbations, and allergic status [serum total immunoglobulin E (IgE) levels, serum specific allergen testing], when available. The present study was approved by the local Ethics Committee.

### Measurements of Spirometry

Spirometry was performed by trained technicians using a pneumotachograph-type spirometer (MasterScreen Pneumo, Jaeger, Hoechberg, Germany), according to the ATS/ERS standards adapted for children (14, 15). For quality control, all the recruited tests were reviewed and selected by two investigators according to the recommendations (14, 15), in order to ensure that the results were valid. Subjects with only one acceptable measurement were excluded. The Global Lung Function Initiative (GLI-2012) reference equations for North East Asians (16) were used to calculate the predicted values and z-scores for the FEV_1_, FVC, FEV_1_/FVC ratio, FEF_25−75%_, and instantaneous forced expiratory flow at 75% of FVC (FEF_75%_). The GLI reference values were computed for each subject using the GLI-2012 Desktop Software for Data Sets Version 1.3.4, which is available on a website: https://www.ers-education.org/media/media.aspx?idMedia=266701&rOk=1. As recommended by ATS/ERS (1), the 5th percentile of the reference population was used as lower limits of normal (LLN), which is also a z-score of −1.64. Abnormal baseline values, which were expressed as z-scores, were defined as smaller than −1.64. According to the values of FEV_1_, FVC, and FEV_1_/FVC, two measurement date-matched groups were defined: restricted (*n* = 66), subjects had a solely reduced FVC and normal FEV_1_/FVC; obstructed (*n* = 143), subjects had reduced FEV_1_/FVC and FEV_1_ with normal FVC. For the purpose of the present study, restricted was referred to as having a reduced FVC spirometry, while obstructed was referred to as a typical obstruction group. Patients with restricted were all children who met the restricted criteria during the 3-year period from 2015 to 2018. To eliminate the influence of different operators on quality control, which cannot be checked retrospectively, the date of measurement was matched between two groups. Patients with obstructed were those measured before or after the selected restricted patient on the same day. The percentage of back extrapolated volume (EV) to FVC was used to assess the quality control for the start of the test. ET was defined as the time from the start of exhalation to the end of exhalation or the start of next inhalation (14, 15). The volume-time curves were recalculated by Engauge Digitizer (version 12.1) to identify the start point of exhalation plateau, which was known as no change in volume (0.025 L) for 1 s on the volume-time curve. ET was divided into effective ET (ETe) and plateau ET (ETp) by the start point of exhalation plateau (Supplementary Figure 1A). If no exhalation plateau can be defined, the exhalation time of last 0.025 L was defined as ETp (Supplementary Figure 1B). Slow vital capacity (SVC) was measured before FVC and FEV_1_. SVC was the maximum VC during slow deep inspiration and expiration at least three times.

### Measurements of Bronchodilation Tests

Short- and long-acting bronchodilators were withheld for at least 8 and 12 h, respectively, before the tests. FEV_1_, FVC, and FEF_25−75%_ reversibility was assessed based on the percentage of the predicted value. In order to further explore the causes of reduced FVC, restricted was divided into two subgroups, according to the change in FVC with bronchodilation (%ΔFVC). “Restricted, +BD” was defined as a %ΔFVC of 10% or greater, while “restricted, –BD” was defined as a %ΔFVC of <10%. A cut-off point of 10% for predicting the change in FVC was selected, which may reflect bronchodilator reversibility in asthmatic children (9, 17).

### Measurements of Fractional Exhaled Nitric Oxide

Before the lung function test, fractional exhaled nitric oxide (FeNO) was measured with an online collection apparatus using an electrochemical device (NIOX MINO, Aerocrine, Solna, Sweden), according to ATS/ERS guidelines (18), as previously described (19).

### Statistical Analysis

Normally distributed variables were expressed as mean ± standard deviation (SD) and compared between groups with two-sample *t*-test. Non-normally distributed variables were presented as medians (interquartile ranges) and compared using the Mann-Whitney *U*-test. Categorical variables were expressed in percentages and compared with the chi-square test (or by Fisher's exact test, in case of expected frequencies of less than five). The relationships between continuous variables were explored using Pearson's correlations. An exploratory analysis was performed to identify variables associated with restricted. A logistic regression model was constructed with a restricted as the dependent variable and the obstructed as the reference category. Tests were also conducted for multicollinearities between variables. Independent variables with significant between-group difference or clinical significance, related to demographics, asthma status, allergen sensitization, FeNO, total IgE, and bronchodilation tests (BDTs), were included in the model and removed in a stepwise manner, minimizing the Akaike information criterion in the final model. Two-tailed *P*-values of < 0.05 was considered statistically significant. The statistical analysis was performed using the IBM SPSS Statistics, version 20 (IBM, Armonk, NY, USA).

## Results

### Demographic and Clinical Characteristics

Table 1 lists the demographic and clinical characteristics of these patients. The present study retrospectively evaluated 66 asthmatic children (16 boys and 50 girls) with restricted and 143 age-matched asthmatic subjects (43 boys and 100 girls) with obstructed. There were no statistically significant differences between restricted and obstructed with respect to age, gender, weight, height, and body mass index (BMI). Furthermore, there were no statistically significant between-group differences with regard to age at diagnosis, asthma duration, treatment duration, exacerbations, FeNO value, serum total IgE level, and the sensitization to mites, foods, molds, pets, and roaches. However, patients with restricted had significantly more sensitizations to plants, when compared with patients with obstructed (38.0 vs. 22.2%, respectively; *P* < 0.05).

**Table 1:** Demographic and clinical characteristics of the study subjects.

**Characteristics**	**All subjects (*n* = 209)**	**“Restricted” (*n* = 66)**	**“Obstructed” (*n* = 143)**	***P-*value**
Age at lung function test (years)^b^	8.2 (6.4, 10.1)	8.8 (5.9, 10.9)	8.0 (6.5, 9.8)	0.420
Gender (% male)	150/59 (71.8%)	50/16 (75.8%)	100/43 (69.9%)	0.384
Weight (kg)^b^	30.2 (25.0, 42.8)	30.0 (24.0, 40.8)	31.0 (25.0, 43.0)	0.413
Height (cm)	136.3 ± 15.3	137.0 ± 18.0	135.9 ± 14.0	0.678
BMI (kg/m^2^)^b^	17.1 (15.1, 20.7)	16.7 (14.7, 19.2)	17.4 (15.1, 21.2)	0.190
Age at asthma diagnosis (years)^a^, ^b^	6.0 (5.0, 8.0)	5.0 (4.0, 8.5)	7.0 (5.0, 8.0)	0.105
Asthma duration (years)	0.50 (0.00, 2.54)	0.96 (0.00, 3.52)	0.42 (0.00, 2.00)	0.175
With treatment (%)	87/122 (41.6%)	30/36(45.5%)	57/86(39.9%)	0.446
Treatment duration (months)^b^	8.0 (3.0, 14.0)	6.00 (2.00, 12.0)	8.0 (3.0, 17.5)	0.145
Exacerbations (%)	22/187 (10.5%)	5/61 (7.6%)	17/126 (11.9%)	0.345
Categories (% positive)	(*n* = 149)	(*n* = 50)	(*n* = 99)	
Mites^a^	68/81 (45.6%)	27/23 (54.0%)	41/58 (41.4%)	0.145
Foods^a^	67/82 (45.0%)	25/25 (50.0%)	42/57 (42.4%)	0.380
Molds^a^	51/98 (34.2%)	18/32 (36.0%)	33/66 (33.3%)	0.746
Pets^a^	21/128 (14.1%)	7/43 (14.0%)	14/85 (14.1%)	0.981
Plants^a^	41/108 (27.5%)	19/31 (38.0%)	22/77 (22.2%)	0.042
Roaches^a^	5/144 (3.4%)	2/48 (4.0%)	3/96 (3.0%)	1.000^c^
FeNO (ppb)^a^, ^b^	18.0 (11.8, 33.0) (*n* = 206)	18.0 (12.0, 32.0) (*n* = 66)	19.0 (11.0, 35.0) (*n* = 140)	0.951
Total IgE level (kU/L)^a^, ^b^	218.2 (90.8, 463.8) (*n* = 151)	228.4 (140.3, 564.5) (*n* = 51)	203.7 (80.3, 416.0) (*n* = 100)	0.160

*Except as noted, continuous variables were expressed as mean ± standard deviation (SD), and the P-values were obtained using the two-sample t-test. The categorical variables were expressed in percentage, and the P-values were determined using the chi-square test. BMI, body mass index, determined as the weight in kilograms divided by the square of the height in meters; FeNO, fractional exhaled nitric oxide*.

a *Due to incomplete data, the sample size was given in the table*.

b *Expressed in median (interquartile range), the P-values were obtained using the Mann-Whitney U test*.

c *The P-values were obtained using the Fisher's exact test*.

### Lung Function Analysis

**Figure 1:**
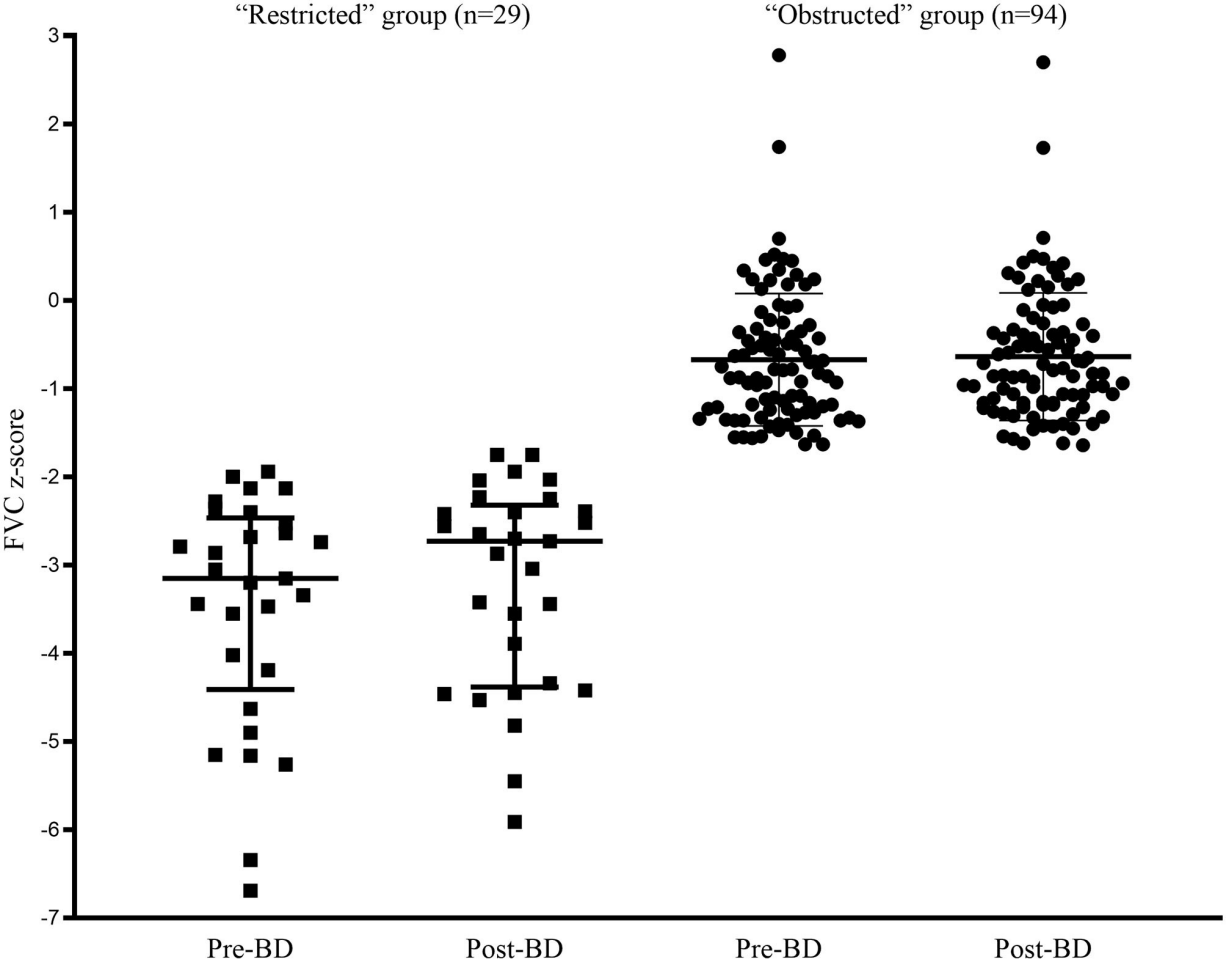
Pre-BD and post-BD FVC in “restricted” and “obstructed” groups.

**Figure 2:**
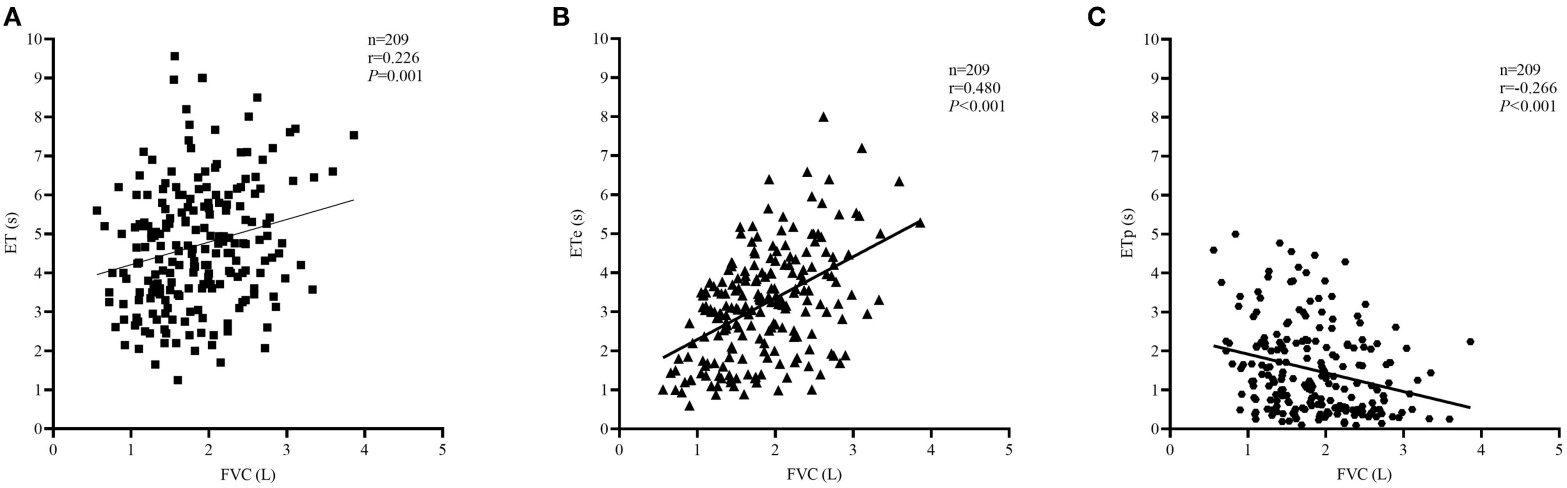
Correlations between ET, ETe, ETp, and FVC in the whole subjects. The correlation coefficients were obtained by Pearson's correlation. **(A)** Changes in ET related to FVC values. **(B)** Changes in ETe related to FVC values. **(C)** Changes in ETp related to FVC values.

**Figure 3:**
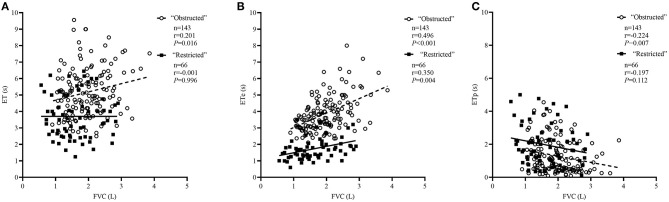
Correlations between ET, ETe, ETp, and FVC in the “obstructed” and “restricted” group separately. The correlation coefficients were obtained by Pearson's correlation. **(A)** Changes in ET related to FVC values. **(B)** Changes in ETe related to FVC values. **(C)** Changes in ETp related to FVC values.

The comparison of spirometric results between these two groups are presented in Table 2. Compared with obstructed subjects, restricted subjects had significantly higher predicted values and z-scores for FEV_1_, FEF_25−75%_, and FEF_75%_, lower levels of bronchodilator response measured by FEV_1_ and FEF_25−75%_, and higher values of SVC/FVC (%) (*p* < 0.05). Patients with restricted trended to have a higher %ΔFVC (*P* = 0.057) when compared with those with obstructed (Figure 1). With regard to the quality control parameters, patients with restricted had significantly higher EV/FVC (%), shorter ET, shorter ETe, and longer ETp, when compared with those with obstructed (*P* < 0.001). In the whole subjects, ET (*r* = 0.226, *P* = 0.001; Figure 2A), ETe (*r* = 0.480, *P* < 0.001; Figure 2B), and ETp (*r* = −0.266, *P* < 0.001; Figure 2C) were significantly associated with FVC. In the obstructed group, ET (*r* = 0.201, *P* = 0.016; Figure 3A), ETe (*r* = 0.496, *P* < 0.001; Figure 3B), and ETp (*r* = −0.224, *P* = 0.007; Figure 3C) was significantly associated with FVC. In the restricted group, only ETe (*r* = 0.350, *P* = 0.004; Figure 3B) was significantly associated with FVC but not ET (*r* = −0.001, *P* = 0.996; Figure 3A) and ETp (*r* = 0.197, *P* = 0.112; Figure 3C).

**Table 2:** Spirometry of the study subjects.

**Items**	**All subjects (*n* = 209)**	**“Restricted” (*n* = 66)**	**“Obstructed” (*n* = 143)**	***P-*value**
**FEV**_**1**_				
% Predicted^b^	76.2 (71.7, 80.6)	78.4 (69.5, 84.6)	75.7 (72.0, 79.0)	<0.001
z-score^b^	−2.58 (−3.08, −2.11)	−2.33 (−3.14, −1.65)	−2.58 (−3.02, −2.26)	0.036
%Δ^b^	12.2 (6.2, 22.0) (*n* = 123)	6.6 (2.3, 11.2) (*n* = 29)	15.3 (9.7, 23.1) (*n* = 94)	<0.001
**FVC**				
% Predicted^b^	89.7 (81.5, 95.0)	77.3 (70.3, 80.8)	92.7 (89.3, 97.0)	<0.001
z-score^b^	−1.23 (−2.27, −0.63)	−2.72 (−3.64, −2.30)	−0.88 (−1.24, −0.36)	<0.001
%Δ	5.0 ± 7.8 (*n* = 123)	7.4 ± 9.1 (*n* = 29)	4.2 ± 7.3 (*n* = 94)	0.057
**FEV**_**1**_**/FVC**				
Ratio	76.5 ± 9.5	88.8 ± 2.7	70.9 ± 5.1	<0.001
% Predicted	86.8 ± 12.0	102.0 ± 7.0	79.8 ± 5.8	<0.001
z-score	−1.80 ± 1.82	0.48 ± 1.46	−2.85 ± 0.60	<0.001
**FEF**_**25–75%**_				
% Predicted	54.0 ± 20.1	74.4 ± 23.2	44.7 ± 8.2	<0.001
z-score	−2.45 ± 1.11	−1.32 ± 1.19	−2.97 ± 0.55	<0.001
%Δ	40.0 ± 31.6 (*n* = 123)	19.12 ± 27.2 (*n* = 29)	46.4 ± 30.2 (*n* = 94)	<0.001
**FEF**_**75%**_				
% Predicted	48.2 ± 24.2	71.6 ± 29.0	37.4 ± 9.9	<0.001
z-score	−2.51 ± 1.24	−1.30 ± 1.32	−3.06 ± 0.70	<0.001
SVC/FVC (%)^a^, ^b^	102.0 (100.8, 104.1) (*n* = 64)	102.7 (1.010, 106.3) (*n* = 30)	101.6 (100.6, 103.7) (*n* = 34)	0.028
ET (s)^b^	4.5 (3.5, 5.7)	3.4 (2.4, 4.4)	4.9 (4.0, 6.0)	<0.001
ETe (s)^b^	3.2 (2.1, 4.0)	1.6 (1.3, 2.0)	3.6 (3.1, 4.4)	<0.001
ETp (s)^b^	0.5 (1.2, 2.2)	1.7 (1.0, 2.6)	1.0 (0.5, 2.1)	<0.001
EV/FVC (%)^b^	2.02 (1.55, 2.69)	2.73 (2.02, 3.40)	1.78 (1.43, 2.29)	<0.001

*Except as noted, continuous variables were expressed as mean ± standard deviation (SD), and the P-values were obtained using the two-sample t-test. FVC, forced vital capacity; %Δ, bronchodilator response, expressed as the percent change from baseline; FEV_1_, forced expiratory volume in 1 s; FEF_25−75%_, mean forced expiratory flow between 25 and 75% of FVC; FEF_75%_, instantaneous forced expiratory flow at 75% of FVC; SVC, slow vital capacity; ET, exhalation time; ETe, effective ET; ETp, plateau ET;EV, back extrapolated volume*.

a *Due to incomplete data, the sample size was given in the table*.

b *Expressed in median (interquartile range), the P-values were obtained using the Mann-Whitney U-test*.

### Factors Associated With Restricted

**Figure 4:**
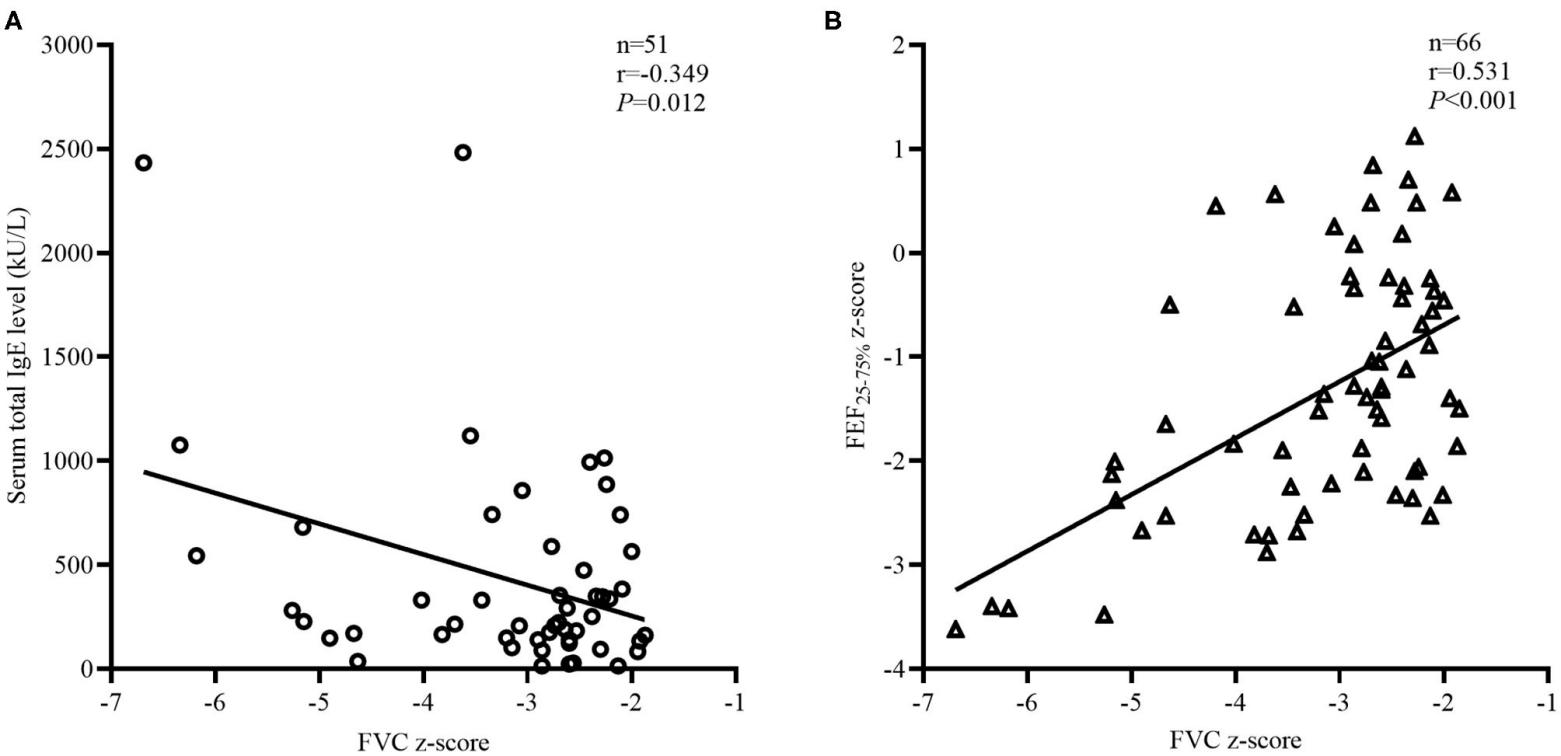
Factors related to FVC z-score in the “restricted” group. The correlation coefficients were obtained by Pearson's correlation. **(A)** Changes in total IgE related to the FVC z-score values. **(B)** Changes in FEF_25−75%_ z-score related to the FVC z-score values.

Logistic regression methods were used to explore the factors associated with restricted. There were significant multicollinearities among variables measured using the variance inflation factor method. After excluding variables with severe multicollinearities and cases with more than one missing variable, the logistic regression in 29 restricted and 94 obstructed subjects with BDTs, revealed that smaller %ΔFEF_25−75%_ and higher %ΔFVC were risk factors associated with reduced FVC (Table 3). Variables that did not contribute significantly to the model included age, BMI, current asthma status, asthma duration, treatment duration, and FeNO. Every 1% increase of %ΔFEF_25−75%_ increased the risk of being restricted by 0.95 (95% CI: 0.93–0.98, *P* < 0.001). Every 1% increase of %ΔFVC increased the risk of being restricted by 1.12 (95% CI: 1.04–1.22, *P* = 0.005). In the restricted group, the total IgE (*n* = 51, *r* = −0.349, *P* = 0.012; Figure 4A) and FEF_25−75%_ z-score (*n* = 66, *r* = 0.531, *P* < 0.001; Figure 4B) were significantly associated with the FVC z-score values but not with age, BMI, FeNO, SVC/FVC (%), %ΔFEF_25−75%_, %ΔFVC, or EV/FVC (%) (*P* > 0.05).

**Table 3:** Logistic regression analysis of predictors of “restricted” in the whole subjects.

**Variables**	**OR**	**95% CI (lower-upper)**	***P*-value**
%ΔFEF_25−75%_	0.95	0.93–0.98	<0.001
%ΔFVC	1.12	1.04–1.22	0.005

*CI, confidence interval; OR, odds ratio; %Δ, bronchodilator response, expressed as the percent change from baseline; FVC, forced vital capacity; FEF_25−75%_, mean forced expiratory flow between 25 and 75% of FVC*.

### BDTs of Restricted

**Figure 5:**
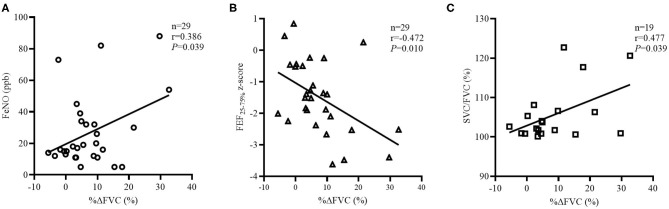
Factors related to %ΔFVC in the “restricted” group with BDTs. The correlation coefficients were obtained by Pearson's correlation. **(A)** Changes in FeNO related to %ΔFVC values. **(B)** Changes FEF_25−75%_ z-score related to %ΔFVC values. **(C)** Changes in SVC/FVC (%) related to %ΔFVC values.

Among the 66 subjects with restricted, the BDTs were available for 29 subjects, and the characteristics are summarized in Supplementary Table 1. No statistically significant between-group differences were observed in the demographic and clinical characteristics. However, restricted, +BD had significantly more sensitizations to foods, when compared with restricted, –BD (87.5 vs. 35.71%, respectively; *P* < 0.05). Compared with restricted, –BD, restricted, +BD had significantly lower predicted values and z-scores for FEF_25−75%_ and FEF_75%_ but had similar FEV_1_, FVC, %ΔFEV_1_, %ΔFEF_25−75%_, EV/FVC (%), ET, ETe, and ETp (Supplementary Table 2). However, restricted, +BD trended to have a higher SVC/FVC (%) (106.6 vs. 101.9, *P* = 0.083), even though the between-group difference was minimally significant (Supplementary Table 2). In the subgroup of patients with restricted and BDTs, FeNO (*n* = 29, *r* = 0.386, *P* = 0.039; Figure 5A), FEF_25−75%_ z-score (*n* = 29, *r* = −0.472, *P* = 0.010; Figure 5B), and SVC/FVC (%) (*n* = 19, *r* = 0.477, *P* = 0.039; Figure 5C) and were significantly associated with %ΔFVC but not with age, BMI, IgE, FeNO, SVC/FVC (%), FVC z-score, %ΔFEF_25−75%_, EV/FVC (%), ET, ETe, or ETp (*P* > 0.05).

## Discussion

The most important finding of the present study was that asthmatic children with reduced FVC had short ET and short ETe but long ETp. In asthmatic children with reduced FVC, there was significant positive correlation between the FVC and ETe but not ETp which represents FVC effort. The regression analysis for the whole subjects revealed that higher %ΔFVC was risk factors of restricted. Asthmatic children with restricted also had a higher SVC/FVC (%) than those with obstructed, which indirectly suggested small airway impairment or loss of elastic recoil (20, 21). It was also found that ET, ETe, ETp, and EV/FVC (%) were comparable between restricted, +BD and restricted, –BD. These data confirmed the present hypothesis that poor FVC effort does not account for the reduction in FVC in asthmatic children. This was mentioned in the ATS/ERS guidelines (1) as a possible reason, but this has rarely been mentioned in previous studies (7–10).

It is important for subjects to be encouraged to continue to exhale the air at the end of the test to obtain optimal effort. A reasonable FVC effort can be identify by enough ET or the presence of exhalation plateau. Subjects in the obstructed group had enough ET, while the restricted group did not. Fifty percent of children in the restricted group reached ET ≥ 3.4 s with a P25 of 2.4 s, which was shorter than 4.9 and 4.0 s in obstructed group separately. Gochicoa-Rangel et al. (22) showed that 87.2% of 5-to-8-year-old healthy children reached ET ≥ 3 s. So, the restricted group had short ET. Short ET can be due to either small age, poor expiratory effort, decreased lung volume, or premature distal airway closure. Firstly, the age of children in this study was slightly older than those reported by Gochicoa-Rangel et al. (22) and age was not correlated with ET in restricted group, so small age did not seem to be the reason for short ET. Secondly, the present study did not perform plethysmography, which could exclude the real restrictive defect. While all the 180 asthmatic children reported by Mahut et al. (10) had TLC more than 80% of predicted value. Although we cannot prove that decreased lung volume was not the reason of short ET, but it cannot be the main one. In order to further evaluate the relationship between ET and FVC, ET was divided into ETe and ETp by the start point of exhalation plateau on the volume-time curve. ETe is the exhalation time that contributes the most to FVC, which was confirmed by the moderate positive correlation between ETe and FVC in both groups. During ETp, FVC has little increase, but ETp reflects the effort of exhalation at the end of test. Results of present study showed restricted group had longer ETp than the obstructed group, and there was no association between ETp and FVC in the restricted group, suggesting that insufficient exhalation and poor FVC effort are not the reason for short ET and reduced FVC. Another interesting finding was that there was mild negative correlation between ETp and FVC in obstructed group. Airway obstruction causes expiratory flow reduction and more effort and time to exhale, leading to prolonged ETe and shortened ETp. Whereas, in the restricted group, there was no correlation between ETp and FVC. The most possible reason is distal airway closure in the early stage of exhalation, which can shorten ETe and reduce FVC synchronously. Although there is no more airflow after the airway is trapped, the child can continue to exhale, which prolongs ETp and does not increase FVC anymore, leading to no correlation between ET, ETp, and FVC. So, it may be speculated that the shorter ET in the restricted group might be due to the special phenotype of asthma but not the poor FVC effort. The differences in the composition of ET between the two groups suggest that the underlying mechanisms may be different. These present results also revealed a higher EV/FVC (%) in the restricted group. According to the ATS/ERS recommendations (14, 15), EV/FVC (%) can be used to judge the effort of the initial part of forced expiration, which affects the FEV_1_ mostly. The elevated EV/FVC (%) in the restricted group was still in normal range and may be explained by the reduced FVC. Although the present study revealed that poor expiratory effort was not a factor that contributes to the reduction in FVC in asthmatic children, there is still a need to pay attention to quality control in clinic, since the included tests met the ATS/ERS standards (14, 15).

FEF_25−75%_, which was considered to be a measurement of small airway patency (1–3), has been challenged by some physiologists as a “volume-dependent” measure (23). This appeared to be normal in asthmatic children with restricted, while this decreased in the obstructed group. These results are consistent with the results reported by Sorkness et al. (9), in which it has demonstrated that FEF_25−75%_ is primarily a measure of airflow limitation that is highly correlated with the FEV_1_/FVC ratio. However, the FEV_1_/FVC ratio was normal due to the simultaneous decline in FVC and FEV_1_ in the restricted group. The association of the FEF_25−75%_ z-score with the FVC z-score in the restricted group was further evaluated, and a moderate positive correlation was found. Without the further measurements in small airway function (e.g., multiple-breath nitrogen washout, impulse oscillometry, etc.), it could not be determined whether the positive correlation was due to small airway dysfunction or just volume reduction. In the subgroup of patients with restricted and BDTs, there was a significant mild correlation between SVC/FVC (%) and %ΔFVC, and restricted, +BD had a decreased FEF_25−75%_, suggesting that small airway smooth muscle tone may contribute to the closure. Another interesting finding was that there was limitation of fixed FEV_1_/FVC ratio to diagnose airflow obstruction, especially for children. Quanjer et al. (24) showed the pattern of FEV_1_/FVC ratio in healthy children was a fall from 0.90 at age 5 years to 0.86 at age 11 years, followed by a rise to 0.87 in adolescence. The FEV_1_/FVC ratios were similar in two groups in the present study, which is consistent with those reported by Thompson et al. (25). So, the ATS/ERS standards recommend the use of LLN in place of a fixed FEV_1_/FVC ratio.

FeNO is a measure of airway inflammation in asthma, which has been associated with small airway dysfunction (26, 27). In the present study, no correlation was found between FeNO and FVC in the restricted group. These results are consistent with those reported by Mappa et al. (28), in which it was demonstrated that there was no correlation between FeNO levels and FVC, but there was a correlation between FeNO and air trapping, as measured through FRC, RV, and RV/TLC in asthmatic children. The present study did not perform plethysmography, but a mild positive correlation between the FeNO and %ΔFVC values was found. Covar et al. (29) and Coverstone et al. (30) reported that the presence of a bronchodilator response assessed by FEV_1_ in asthmatic children correlated to the FeNO level, suggesting uncontrolled asthma (4, 31). However, Sorkness et al. (9) reported that a reduced FVC or %ΔFVC ≥ 10% suggests greater asthma severity and instability. In the present study, restricted, +BD had shorter asthma durations, but had comparable treatment durations, when compared with restricted, –BD. Serum IgE levels, the sensitization to molds, age at asthma diagnosis, and BMI were identified as important risk factors of difficult-to-treat asthma (32). Similarly, in the present study, when compared with obstructed, restricted had more sensitization to plant allergies. In the restricted group, FVC was negatively mildly correlated with the serum IgE level, and restricted, +BD had more sensitization to food allergies. Therefore, there is reason to speculate that persistent allergen exposure may be one of the causes of reduced FVC in asthmatic children.

The prevalence and underlying causes of the reduced FVC in asthmatic children remain poorly understood. In the restricted group, sensitization to plants may lead to elevated eosinophilic airway inflammation, revealed by positive correlation between FeNO and %ΔFVC values. Although the airway inflammation of asthma affects the entire airway, small airways without cartilage are the main sites of obstruction (4). Asthma is a kind of heterogenetic disease, so does the obstruction of small airway (4). The small airway dysfunction in the restricted and obstructed groups differed, suggested by that restricted group had slightly higher %ΔFVC and higher %ΔFVC increased the risk of being restricted. Air trapping caused by distal airway closure in the early stage of exhalation may be the main reason for the FVC reduction in asthmatic children, as suggested in several studies that used plethysmography (7, 8, 10). The smooth muscle contraction, increased surface tension, and increased volume of intraluminal material caused by the elevated eosinophilic airway inflammation accounted for the initial mechanism of distal airway closure, which may have a good response to ICS (23). As the disease progresses, loss of elastic recoil of the distal airway leading to reduced FVC without bronchodilator reversibility. Reduced airway-parenchymal coupling wall may also occur, leading to dynamic airway closure during a forced expiration, which was observed as an elevated SVC/FVC (%) and significant moderate association between SVC/FVC (%) and %ΔFVC in the restricted group. The premature distal airway closure would make patients unable to voluntarily exhale more volume, leading to shorter ET and increased RV, as assessed by plethysmography (7, 8, 10). For obstructed, the airflow limitation of the small airways would lead to a normal or longer ET, as revealed by these present results.

The present functional study had several limitations. Firstly, our results may be limited by the retrospective design. Although data are meticulously recorded in our clinic, they were collected from the electronic medical records and some of them were missing. Although we retrospectively analyzed asthma duration, treatment duration, and exacerbations to assess the severity of asthma, if the level of asthma control could be assessed, the causes for FVC reduction will be better understood. Due to the retrospective analysis, the higher frequency of BDTs in the obstructed group than the restricted group (94/143 vs. 29/66, *P* = 0.003) might introduce a selection bias. Secondly, the present study did not perform plethysmography, which could exclude the real restrictive defect. Plethysmography is often too complicated for children and is not available in most pediatric departments. Thirdly, subjects with both reduced FVC and reduced FEV_1_/FVC ratio were not included, whose obstructions of airway may be more complex, making it difficult to analyze the reason of reduced FVC. The aim of the present study was to determine the contribution of FVC effort to FVC reduction. Hence, a group with solely reduced FVC was designed. Regardless of whether isolated reduced FVC is associated with lower levels of asthma control, the further decline in lung function and/or refractory asthma are important questions that were beyond the scope of this functional retrospective study. More work should be done to know the prevalence of this special pattern of asthma in children, because the mechanism of small airway dysfunction of children may be different from adults. Further prospective evaluations for asthma control level and long-term follow-ups, including oscillometry, may help to determine the pathophysiologic role of distal airway dysfunction in these children.

In summary, short ETe and long ETp suggested that poor FVC effort does not account for the FVC reduction in asthmatic children. The short ET and high SVC/FVC (%) may be caused by premature distal airway closure and are the characteristics of reduced FVC in routine spirometry. FeNO, BDTs, serum IgE level, and serum-specific allergen tests may be helpful in identifying the possible risk factors in asthmatic children with reduced FVC.

## Data Availability Statement

The raw data supporting the conclusions of this article will be made available by the authors, without undue reservation.

## Ethics Statement

The studies involving human participants were reviewed and approved by Shengjing Hospital of China Medical University. Written informed consent from the participants' legal guardian/next of kin was not required to participate in this study in accordance with the national legislation and the institutional requirements.

## Author Contributions

YF and YS conceived and designed the study. YF and QY were responsible for the collection and analysis of the experimental data. YF interpreted the data and drafted the manuscript which was revised by all authors. All authors read and approved the final manuscript.

## Conflict of Interest

The authors declare that the research was conducted in the absence of any commercial or financial relationships that could be construed as a potential conflict of interest.
