# Supplementary material for: “Poor Effort” Does Not Account for Reduced Forced Vital Capacity in Asthmatic Children
Source: Front Pediatr. 2021 May 25;9:596384. doi: 10.3389/fped.2021.596384 (PMC8185061; doi:10.3389/fped.2021.596384)
Supplement: Supplementary file 1 [file Table_1.docx]

Supplementary Material

# Supplementary Tables

## Supplementary Table 1. Demographic and clinical characteristics of subjects with “Restricted” and BDTs.

| **Characteristics** | **"Restricted, +BD"**  **(*n*=10)** | **"Restricted, -BD"**  **(*n*=19)** | ***P-*value** |
| --- | --- | --- | --- |
| Age at lung function test (years)^b^ | 7.3 (4.9, 11.1) | 9.5 (6.6, 10.7) | 0.377 |
| Gender (% male) | 7/3 (70.0%) | 13/6 (68.4%) | 1.000^c^ |
| Weight (kg) ^b^ | 27.6 (16.6, 47.6) | 29.0 (22.4, 48.6) | 0.484 |
| Height (cm) | 132.2±24.3 | 140.1±20.0 | 0.355 |
| BMI (kg/m^2^)^b^ | 17.2 (14.4, 19.9) | 16.7 (13.4, 18.8) | 0.735 |
| Age at asthma diagnosis (years)^a b^ | 7.0 (4.0, 11.0) | 7.0 (4.0, 8.0) | 0.458 |
| Asthma duration (years) | 0.00 (0.00, 0.23) | 1.00 (0.00, 3.58) | 0.015 |
| Treatment duration (months) | 0.00 (0.00, 0.25) | 1.00 (0.00, 6.00) | 0.085 |
| Current asthma status  (% exacerbations) | 1/9 (10.0%) | 2/17 (10.5%) | 1.000^c^ |
| Categories (% positive) | | | |
| Mites^a^ | 6/2 (75.0%) | 6/8 (42.86%) | 0.204^c^ |
| Foods^a^ | 7/1 (87.5%) | 5/9 (35.71%) | 0.031^c^ |
| Molds^a^ | 3/5 (37.5%) | 6/8 (42.86%) | 1.000^c^ |
| Pets^a^ | 1/7 (12.5%) | 1/13 (7.14%) | 1.000^c^ |
| Plants^a^ | 3/5 (37.5%) | 6/8 (42.86%) | 1.000^c^ |
| Roaches^a^ | 1/7 (12.5%) | 0/14 (0.0%) | 0.364^c^ |
| FeNO (ppb) ^a b^ | 23.0 (9.5, 61.0) | 16.0 (12.0, 32.0) | 0.566 |
| Total IgE level (kU/L) ^a b^ | 512.4 (155.0, 1021.6) (n=8) | 199.6 (86.0, 389.2) (n=14) | 0.219 |

Except as noted, continuous variables were expressed as mean ± standard deviation (SD), and the *P*-values were obtained using the two-sample *t*-test. The categorical variables were expressed in percentage, and the *P*-values were determined using the chi-square test. BMI: body mass index, determined as the weight in kilograms divided by the square of the height in meters; FeNO: fractional exhaled nitric oxide. ^a^ Due to incomplete data, the sample size was given in the table; ^b^ Expressed in median (interquartile range), the *P*-values were obtained using the Mann-Whitney *U*-test; ^c^ The *P*-values were obtained using the Fisher’s exact test.

## Supplementary Table 2. Spirometry of subjects with “Restricted” and BDTs.

| **Items** | **"Restricted, +BD"**  **(*n*=10)** | **"Restricted, -BD"**  **(*n*=19)** | ***P-*value** |
| --- | --- | --- | --- |
| FEV_1_ |  |  |  |
| % Predicted^b^ | 72.4 (56.6, 77.0) | 77.9 (63.0, 83.8) | 0.104 |
| z-score^b^ | -2.66 (-5.39, -2.05) | -2.73 (-3.12, -1.65) | 0.330 |
| %Δ^b^ | 7.5 (2.7, 18.4) | 6.6 (1.4, 9.9) | 0.308 |
| FVC |  |  |  |
| % Predicted^b^ | 75.6 (55.9, 78.0) | 75.3 (66.3, 80.3) | 0.422 |
| z-score^b^ | -3.20 (-5.53, -2.24) | -3.15 (-1.02, -2.53) | 0.636 |
| %Δ | 16.9±8.5 | 2.4±4.0 | <0.001 |
| FEV_1_/FVC |  |  |  |
| Ratio | 89.8±3.9 | 88.4±3.1 | 0.679 |
| % Predicted | 100.0±8.1 | 103.5±4.6 | 0.231 |
| z-score | 0.09±1.62 | 0.74±0.94 | 0.266 |
| FEF_25-75%_ |  |  |  |
| % Predicted | 55.4±20.1 | 79.3±18.4 | 0.003 |
| z-score | -2.34±1.16 | -1.04±0.90 | 0.002 |
| %Δ | 19.7±40.1 | 18.9±18.6 | 0.952 |
| FEF_75%_ |  |  |  |
| % Predicted | 49.4±25.6 | 76.1±22.2 | <0.001 |
| z-score | -2.43±1.29 | -1.02±0.93 | 0.002 |
| SVC/FVC (%)^a b^ | 106.6 (100.9, 120.6) (n=7) | 101.9 (100.8, 103.9) (n=12) | 0.083 |
| ET (sec)^b^ | 3.8 (2.7, 5.4) | 3.3 (2.2, 4.3) | 0.330 |
| ETe (sec)^b^ | 1.3 (1.2, 1.7) | 1.6 (1.2, 1.9) | 0.456 |
| ETp (sec)^b^ | 2.6 (1.5, 3.6) | 1.6 (0.8, 2.3) | 0.115 |
| EV/FVC (%)^b^ | 2.73 (1.70, 3.01) | 2.71 (2.48, 3.89) | 0.247 |

Except as noted, continuous variables were expressed as mean ± standard deviation (SD), and the *P*-values were obtained using the two-sample *t*-test. FVC: forced vital capacity; %Δ: bronchodilator response, expressed as the percent change from baseline; FEV_1_: forced expiratory volume in 1 second; FEF_25-75%_: mean forced expiratory flow between 25% and 75% of FVC; FEF_75%_: instantaneous forced expiratory flow at 75% of FVC; SVC: slow vital capacity; ET: exhalation time; ETe, effective ET; ETp, plateau ET; EV: back extrapolated volume. ^a^ Due to incomplete data, the sample size was given in the table; ^b^ Expressed in median (interquartile range), the *P*-values were obtained using the Mann-Whitney *U*-test.

# Supplementary Figures

**Supplementary Figure 1.** Volume-time curves of a subject with “Restricted” and a subject with “Obstructed”. **(A)** ET was divided into effective ET (ETe) and plateau ET (ETp) by the start point (point a) of exhalation plateau. ETe=1.89 sec. ETp=2.16 sec. **(B)** No exhalation plateau could be defined, the exhalation time of last 0.025 L was defined as ETp. ETe=5.79 sec. ETp=0.67 sec.
