# Supplementary figures and images for: “Poor Effort” Does Not Account for Reduced Forced Vital Capacity in Asthmatic Children
Source: Front Pediatr. 2021 May 25;9:596384. doi: 10.3389/fped.2021.596384 (PMC8185061; doi:10.3389/fped.2021.596384)

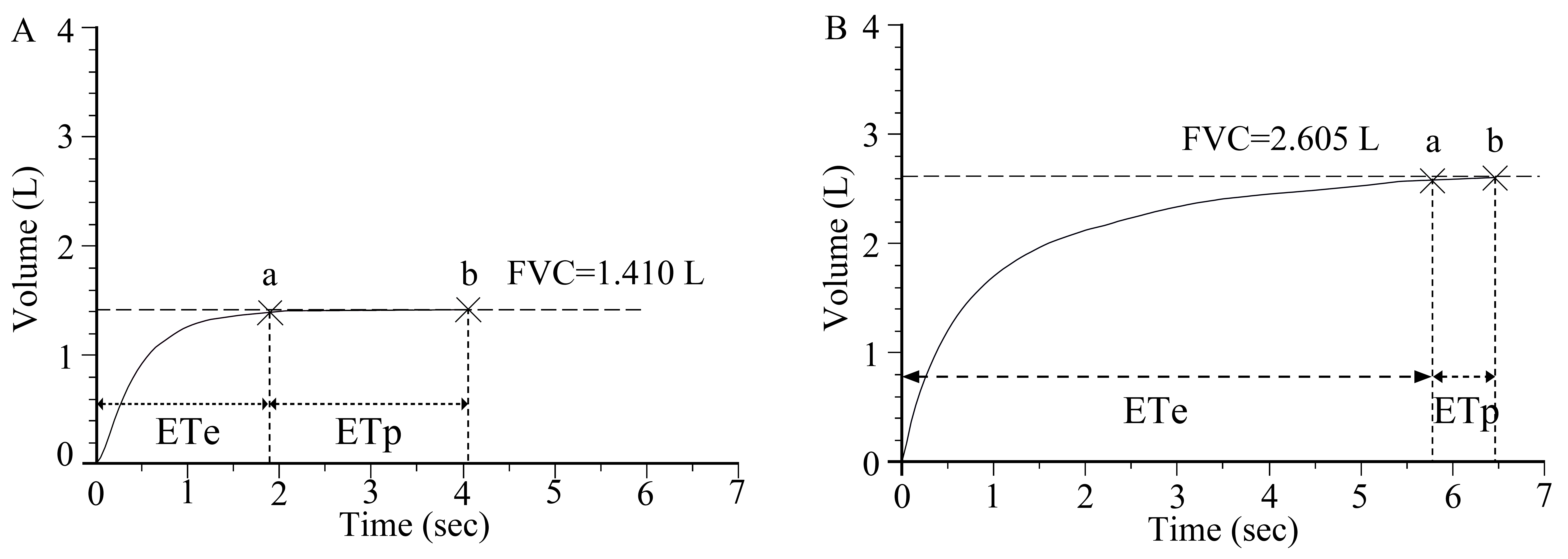

Supplement: Supplementary file 2 [file Image_1.TIF]
